# Factors associated with an increase in tobacco use and alcohol drinking during the COVID-19 pandemic: A cross-sectional study of data from 105 countries

**DOI:** 10.18332/tid/157205

**Published:** 2023-01-27

**Authors:** Mir Faeq A. Quadri, Joanne Lusher, Morenike O. Folayan, Maha El Tantawi, Ariel A. Zuñiga, Brandon Brown, Nourhan M. Aly, Joseph C. Okeibunor, Giuliana Florencia, Mohammed Jafer, Eshrat Ara, Kessketlen A. Miranda, Nuraldeen M. Al-Khanati, Passent Ellakany, Balgis Gaffar, Anthonia O. Ishabiyi, Abeedha T. Khan, Zumama Khalid, Folake B. Lawal, Ntombifuthi Nzimande, Anas Shamala, Ala’a B. Al-Tammemi, Bamidele E. Osamika, Muhammad A. Yousaf, Jorma I. Virtanen, Annie L. Nguyen

**Affiliations:** 1 Dental Public Health Division, College of Dentistry, Jazan University, Jazan, Saudi Arabia; 2 Department of Oral Health Sciences, School of Dentistry, University of Washington, Seattle, United States; 3 Regent’s University London, London, United Kingdom; 4 Department of Child Dental Health, Obafemi Awolowo University, Ile-Ife, Nigeria; 5 Department of Pediatric Dentistry and Dental Public Health, Faculty of Dentistry, Alexandria University, Alexandria, Egypt; 6 University of Sierra Sur, Oaxaca, Mexico; 7 Department of Social Medicine, Population and Public Health, University of California Riverside School of Medicine, Riverside, United States; 8 World Health Organization - Regional Office for Africa, Brazzaville, Republic of Congo; 9 Department of Health Promotion, Maastricht University, Maastricht, The Netherlands; 10 Department of Psychology, Government College for Women, Cluster University of Srinagar, Srinagar, India; 11 Research Centre in Physical Activity, Health and Leisure, University of Porto, Porto, Portugal; 12 Department of Oral and Maxillofacial Surgery, Faculty of Dentistry, Syrian Private University, Damascus, Syria; 13 Department of Substitutive Dental Sciences, College of Dentistry, Imam Abdulrahman Bin Faisal University, Dammam, Kingdom of Saudi Arabia; 14 Department of Preventive Dental Sciences, College of Dentistry, Imam Abdulrahman Bin Faisal University, Dammam, Kingdom of Saudi Arabia; 15 Centre for Rural Health, School of Nursing and Public Health, University of KwaZulu-Natal, Durban, South Africa; 16 School of Biological Sciences, University of the Punjab, Lahore, Pakistan; 17 Department of Health Sciences, University of Genova, Genova, Italy; 18 Department of Periodontology and Community Dentistry, University of Ibadan, Ibadan, Nigeria; 19 Department of Economic and Human Geography, University of Szeged, Szeged, Hungary; 20 Department of Preventive and Biomedical Science, Faculty of Dentistry, University of Science and Technology, Sana'a, Yemen; 21 Migration Health Division, International Organization for Migration, United Nations Migration Agency, Amman, Jordan; 22 Applied Science Research Center, Applied Science Private University, Amman, Jordan; 23 Department of Psychology, Lead City University, Ibadan, Nigeria; 24 Institute of Zoology, University of the Punjab, Lahore, Pakistan; 25 Department of Biology, Virtual University of Pakistan, Lahore, Pakistan; 26 Faculty of Medicine, University of Turku, Turku, Finland; 27 Department of Family Medicine, Keck School of Medicine, University of Southern California, Los Angeles, United States

**Keywords:** alcohol use, COVID-19, risk factors, tobacco use, global data

## Abstract

**INTRODUCTION:**

The COVID-19 pandemic has inevitably led to monumental challenges, and alcohol drinking and tobacco use have unlikely been spared. This cross-sectional survey reports on factors associated with an increase in alcohol drinking and tobacco use during the COVID-19 pandemic.

**METHODS:**

An online survey conducted in 2020, generated data from 14899 adults residing in 105 countries. Dependent variables were changes in alcohol drinking and tobacco use. Independent variables were age, sex, education level, job loss, lost or reduced wages, investment/retirement benefits, interrupted substance addiction care, and income level of the countries. Multilevel logistic regression analysis was computed to explore the associations between dependent and independent variables in adjusted models using the backward stepwise method. The probability of including or excluding a covariate was set at p(in)<0.05 and p(out)>0.1, respectively.

**RESULTS:**

Of the regular alcohol consumers (N=4401), 22.9% reported an increase in their alcohol drinking. Of the regular tobacco users (N=2718), 31% reported an increase in their tobacco use. Job loss (Alcohol: AOR=1.26; Tobacco: AOR=1.32) and lost/reduced wages (Alcohol: AOR=1.52; Tobacco: AOR=1.52) were associated with higher odds of increased alcohol drinking and tobacco use. Many interruptions to addiction care (AOR=1.75) were associated with higher odds of increased alcohol drinking. Whereas no interruption to addiction care was associated with lower odds of increased alcohol drinking (AOR=0.77). Also, none (AOR=0.66) or some (AOR=0.70) interruptions to addiction care were associated with lower odds of increased tobacco use.

**CONCLUSIONS:**

This global survey alludes to the unintended consequences of the current COVID-19 pandemic on alcohol drinking and tobacco use. It is critical that the strategies for emergency responses should include support to ameliorate the impact of financial distress and disruption in substance dependence treatment services.

## INTRODUCTION

Alcohol drinking and tobacco use are global public health concerns. About 10% of the world’s population is alcohol- and tobacco-dependent^1-5^. The COVID-19 pandemic has inevitably led to monumental challenges, and alcohol drinking and tobacco use have unlikely been spared. Evidence from primary studies shows that alcohol sales in the United Kingdom during the COVID-19 lockdown, soared by £160 million in comparison to sales in the previous year, and in the United States, 60% of adults reported an increase in alcohol drinking^6-8^. However, a meta-analysis indicated that the mean change in global alcohol drinking was non-significant and that the changes are moderated by per-capita gross domestic product (GDP)^9^. Current data on the change in alcohol drinking during the pandemic are derived mostly from high-income countries, with reported rates of increase ranging from 21.7% to 72.9%^9^. The identified predictors of change in alcohol drinking include contextual and individual-related factors, like COVID-19-related stress^10^, family income loss^11^, lower education level^12^, and age and sex^13^.

Alcohol drinking and tobacco use are closely linked. People who consume alcohol are more likely to smoke and vice versa^14-16^. Similar to the trend of alcohol drinking, the findings on global tobacco use during the COVID-19 pandemic are also mixed. Over 40% of daily smokers in New Zealand reported an increase in the frequency of smoking during the lockdown when compared to the period before the pandemic^17,18^. The increases were observed mostly in daily smokers and were associated with loneliness and isolation^17^. In contrast, a study from China reported that smokers significantly reduced the number of cigarettes during the current pandemic^8^. This reduction was more evident in women and less prominent among individuals with long smoking histories and those residing in urban areas^8^.

The collateral consequences of the COVID-19 pandemic are far from conclusive. The findings on change in alcohol drinking and tobacco use during this pandemic are mixed and the data are mostly available from high-income countries. It is probable that changes vary between high-, middle-, and lowincome countries because of differences in local supply chains, production and distribution of goods, and purchasing power. Obtaining data from both developed and developing countries to identify changes in alcohol drinking and tobacco use during the COVID-19 pandemic, and determining the plausible risk factors associated with the shift could aid in developing informed public health strategies, specially to support the people with alcohol and tobacco dependence problems. This will also assist in managing the magnitude of the alcohol- and tobacco-related problems that may differ between high-, middle-, and low-income countries^19^. Therefore, the objectives of this study were firstly, to determine the proportion of people across several countries who reported a change in alcohol drinking and tobacco use during the COVID-19 pandemic, and secondly, to determine the factors associated with the increase in alcohol drinking and tobacco use.

## METHODS

### Study design and participant recruitment procedure

Data for this study were drawn from a cross-sectional dataset that included 14899 participants from 105 countries (Supplementary file). This global study used a convenience sampling technique to recruit participants to take an online survey (Survey Monkey®). The survey was launched on 29 June 2020 and remained open until 31 December 2020. Respondents aged ≥18 years who were able to read, had access to the internet, and consented to participate, were recruited. Restrictions were applied to survey settings so that each participant could only take the survey once. Participants were able to edit their answers freely until they chose to submit. Identifiers were not collected to ensure that responses were anonymous.

Study participants were recruited through crowdsourcing using various social media platforms (Facebook, Twitter, and Instagram), WhatsApp group or email. The survey was preceded by an introduction about the study team, study objectives, and time needed to complete the questionnaire. This was followed by informed consent assuring participants of the confidentiality of their responses and emphasizing that their participation was voluntary. Only participants who responded affirmatively to the consent could proceed to the survey.

### Instrument validation and study variables

The data collection tool was developed for a study targeting responders across 105 countries (collaboration could not be established in remaining 88 countries). The initial questionnaire was developed through consensus using evidence from published literature and adapted to fit the content domain. The clarity and comprehensiveness of the final version of the questionnaire was also considered. The content representation of each item in the questionnaire was decided based on sensitivity^20^. Further details on survey development are published elsewhere^21^. The questionnaire was available in English, French, Spanish, Portuguese and Arabic. Non-English versions were translated into the respective languages and then back translated to check for discrepancies.

The dependent variables considered were: 1) increase in alcohol drinking, and 2) tobacco use. These variables were measured independently by asking whether participants experienced a change in alcohol drinking and tobacco use during the pandemic (increase, decrease, no change, not applicable). Those who responded ‘not applicable’ were treated as non-consumers of alcohol and/or tobacco while those that reported an increase, decrease or no change were treated as consumers.

The independent variables were: age, sex assigned at birth, education level (no education, primary, secondary, or university level), job loss (yes/no), lost or reduced wages (yes/no), investment/retirement benefits (yes/no), interrupted substance addiction care (not at all, somewhat, a lot, and do not receive care), and income-level. Income-level of the countries was classified according to gross national income per capita (US$ in 2019) by the World Bank^22^ : low-income countries (LICs) ≤1035, lower middle-income countries (LMICs) 1036–4045, upper middle-income countries (UMICs) 4046–12535, and high-income countries (HICs) ≥12536. Income-level was included because income can significantly impact substance use and the level and quality of healthcare received by citizens^23^.

### Statistical analysis

Only respondents with complete data were included in analyses. Statistical analyses were conducted using SPSS software version 23.0 (IBM Corp., Armonk, N.Y., USA). Descriptive analyses were conducted on outcome and independent variables. T-test and chi-squared test were computed depending on the nature of the variables. Multilevel logistic regression analysis was used in exploring the associations between outcome and independent variables separately, then simultaneously in adjusted models. Binary logistic regressions were conducted to determine the relationship between outcome and independent variables. Next, two independent multiple logistic regression analyses were carried out with increased alcohol drinking and tobacco use as outcomes. Only significant variables from the binary logistic regression analyses were included in the multiple regression analyses. The backward stepwise method was used and the probability of including or excluding a covariate was set at p(in)<0.05 and p(out)>0.1, respectively. Odds ratios (OR) and adjusted odds ratios (AOR) with 95% confidence intervals (CI) are reported. Both sets of multivariable logistic regressions were adjusted for age, sex, and education level. The alpha was set at 0.05 for all analyses.

## RESULTS

The mean age of participants was 35.9 ± 12.9 years and 62.6% were women. The proportions of participants who experienced job loss, lost/reduced wages, and lost investment or retirement benefits in the first wave of the COVID-19 pandemic were 10.1% (n=1503), 22.2% (n=3305), and 7.4% (n=1102), respectively (Table 1).

**Table 1:** Sociodemographic characteristics of the respondents (N=14899)

*Factors*	*Categories*	*n (%)*
**Country income level**	LICs	355 (2.4)
	LMICs	7891 (53.0)
	UMICs	3007 (20.2)
	HICs	3646 (24.5)
**COVID-19 interrupted substance addiction care**	Do not receive care	12157 (81.6)
	Not at all	2086 (14.0)
	Somewhat	510 (3.4)
	A lot	146 (1.0)
**Sex**	Male	5579 (37.4)
	Female	9320 (62.6)
**Age** (years), mean ± SD		35.9 ± 12.9
**Education level**	No education, primary or secondary level	3234 (21.7)
	University level	11665 (78.3)
**Job loss or laid off**	Yes	1503 (10.1)
	No	13396 (89.9)
**Lost or reduced wages**	Yes	3305 (22.2)
	No	11594 (77.8)
**Investment/retirement benefits loss**	Yes	1102 (7.4)
	No	13797 (92.6)

LICs: low-income countries. LMICs: lower middle-income countries. UMICs: upper middle-income countries. HICs: high-income countries. SD: standard deviation.

Among the participants, 4401 (29.5%) reported consuming alcohol and 2718 (18.2%) reported using tobacco (Table 2). The highest proportion of alcohol consumers (38.4%, p<0.001) and tobacco users (36.6%, p<0.001) were from HICs (Table 2). Also, a greater proportion of women were alcohol consumers (56.6%, p<0.001) and a greater proportion of men used tobacco (53.5%, p<0.001). Those who consumed alcohol were older than the non-consumers (37.1 vs 34.6 years, p<0.001), while there was no significant age difference among participants who were tobacco users and non-users (p=0.92).

**Table 2:** Bivariate analyses to assess the factors associated with alcohol drinking and tobacco use during the first wave of the COVID-19 pandemic (N=14899)

*Factors*	*Categories*	*Alcohol non-consumers (N=10498) n (%)*	*Alcohol regular-consumers (N=4401) n (%)*	*p*	*Tobacco non-users (N=12181) n (%)*	*Tobacco regular-users (N=2718) n (%)*	*p*
**Country income level**	LICs	296 (2.8)	59 (1.3)	<0.001	296 (2.4)	59 (2.2)	<0.001
	LMICs	6409 (61.0)	1482 (33.7)		7009 (57.5)	882 (32.5)	
	UMICs	1838 (17.5)	1169 (26.6)		2225 (18.3)	782 (28.8)	
	HICs	1955 (18.6)	1691 (38.4)		2651 (21.8)	995 (36.6)	
**Sex**	Male	3668 (34.9)	1911 (43.4)	<0.001	4124 (33.9)	1455 (53.5)	<0.001
	Female	6830 (65.1)	2490 (56.6)		8057 (66.1)	1263 (46.5)	
**Age** (years)^a^, mean ± SD		34.6 ± 12.7	37.1 ± 13.0	<0.001	35.4 ± 12.9	35.3 ± 12.4	0.92
**Education level**	No education, primary or secondary level	2315 (22.1)	919 (20.9)	0.114	2575 (21.1)	659 (24.2)	<0.001
	University level	8183 (77.9)	3482 (79.1)		9606 (78.9)	2059 (75.8)	
**COVID-19 interrupted substance addiction care**	Do not receive care	8602 (81.9)	3555 (80.8)	<0.001	10184 (83.6)	1973 (72.6)	<0.001
	Not at all	1544 (14.7)	542 (12.3)		1636 (13.4)	450 (16.6)	
	Somewhat	276 (2.6)	234 (5.3)		276 (2.3)	234 (8.6)	
	A lot	76 (0.7)	70 (1.6)		85 (0.7)	61 (2.2)	
**Job loss or laid off**	Yes	947 (9.0)	556 (12.6)	<0.001	1066 (8.8)	437 (16.1)	<0.001
	No	9551 (91.0)	3845 (87.4)		11115 (91.2)	2281 (83.9)	
**Lost or reduced wages**	Yes	2231 (21.3)	1074 (24.4)	<0.001	2620 (21.5)	685 (25.2)	<0.001
	No	8267 (78.7)	3327 (75.6)		9561 (78.5)	2033 (74.8)	
**Investment/retirement benefit loss**	Yes	702 (6.7)	400 (9.1)	<0.001	847 (7.0)	255 (9.4)	<0.001
	No	9796 (93.3)	4001 (90.9)		11334 (93.0)	2463 (90.6)	

LICs: low-income countries. LMICs: lower middle-income countries. UMICs: upper middle-income countries. HICs: high-income countries.

a T-test was computed. Chi-squared test was used for all other analysis. SD: standard deviation.

A significantly greater proportion of participants who consumed alcohol and who used tobacco who had reported that their substance treatment had been interrupted ‘somewhat’ or ‘a lot’, lost their jobs (p<0.001), experienced lost/reduced wages (p<0.001), and lost investment or retirement benefits (p<0.001) when compared to occasional/non-consumers and users of alcohol and tobacco, respectively.

As shown in Table 3, 1006 (22.9%) of the 4401 regular consumers of alcohol reported an increase in alcohol drinking during the pandemic. A significantly greater proportion of participants from HICs reported an increase in alcohol drinking (50.3%, p<0.001). Interruptions to addiction treatment (p<0.001) and lost/reduced wages (p<0.001) were associated with increased alcohol drinking.

**Table 3:** Bivariate analyses to assess factors associated with increased alcohol drinking and increased tobacco use during the first wave of the COVID-19 pandemic (Alcohol: N=4401; Tobacco: N=2718)

*Factors*	*Categories*	*Decrease or no change in alcohol drinking (N=3395) n (%)*	*Increase in alcohol drinking (N=1006) n (%)*	*p*	*Decrease or no change in tobacco use (N= 1876) n (%)*	*Increase in tobacco use (N=842) n (%)*	*p*
**Country income level**	LICs	47 (1.4)	12 (1.2)	<0.001	42 (2.2)	17 (2.0)	<0.001
	LMICs	1224 (36.1)	258 (25.6)		670 (35.7)	212 (25.2)	
	UMICs	939 (27.7)	230 (22.9)		473 (25.2)	309 (36.7)	
	HICs	1185 (34.9)	506 (50.3)		691 (36.8)	304 (36.1)	
**Sex**	Male	1490 (43.9)	421 (41.8)	0.181	988 (52.7)	467 (55.5)	0.25
	Female	1905 (56.1)	585 (58.2)		888 (47.3)	375 (44.5)	
**Age** (years), mean ± SD		37.1 ± 13.1	37.2 ± 12.4	0.938	35.3 ± 12.4	35.4 ± 12.3	0.78
**Education level**	No education, primary or secondary level	714 (21.0)	205 (20.4)	0.654	461 (24.6)	198 (23.5)	0.522
	University level	2681 (79.0)	801 (79.6)		1415 (75.4)	644 (76.5)	
	Do not receive care	2728 (80.4)	827 (82.2)	<0.001	1319 (70.3)	654 (77.7)	0.001
**COVID-19 interrupted substance addiction care**	Not at all	447 (13.2)	95 (9.4)		348 (18.6)	102 (12.1)	
	Somewhat	175 (5.2)	59 (5.9)		171 (9.1)	63 (7.5)	
	A lot	45 (1.3)	25 (2.5)		38 (2.0)	23 (2.7)	
**Job loss or laid off**	Yes	411 (12.1)	145 (14.4)	0.193	290 (15.5)	147 (17.5)	0.053
	No	2984 (87.9)	861 (85.6)		1586 (84.5)	695 (82.5)	
**Lost or reduced wages**	Yes	775 (22.8)	299 (29.7)	<0.001	428 (22.8)	257 (30.5)	<0.001
	No	2620 (77.2)	707 (70.3)		1448 (77.2)	585 (69.5)	
**Investment/retirement loss**	Yes	309 (9.1)	91 (9.0)	0.003	155 (8.3)	100 (11.9)	0.96
	No	3086 (90.9)	915 (91.0)		1721 (91.7)	742 (88.1)	

LICs: low-income countries. LMICs: lower middle-income countries. UMICs: upper middle-income countries. HICs: high-income countries. SD: standard deviation.

Among the 2718 tobacco users, 842 (31%) reported an increase in tobacco use during the pandemic. A significantly greater proportion of participants from UMICs reported an increase in tobacco use (p<0.001). Lost/reduced wages (p<0.001) and loss of investments or retirement benefits (p<0.001) were associated with increased tobacco use.

Table 4 presents the findings from multiple logistic regression analyses with alcohol drinking and tobacco use as separate dependent variables in two independent models. Residing in LMICs (AOR=0.47; 95% CI: 0.40–0.56), UMICs (AOR=0.57; 95% CI: 0.48–0.68), and having no interruptions to addiction care (AOR=0.77; 95% CI: 0.61–0.98) were protective against increased alcohol drinking during the pandemic. Conversely, ‘a lot’ of interruptions to addiction care (AOR=1.75; 95% CI: 1.05–2.91) and lost/reduced wages (AOR=1.52; 95% CI: 1.29–1.79) were associated with higher odds of increased alcohol drinking.

**Table 4:** Multiple regression analyses to determine the factors associated with increase alcohol drinking and tobacco use during the first wave of the COVID-19 pandemic (Alcohol: N=4401; Tobacco: N=2718)

*Factors*	*Categories*	*Increase in alcohol drinking*		*Increase in tobacco use*	
		*AOR (95% CI)*	*p*	*AOR (95% CI)*	*p*
**Country income level**	LICs	0.70 (0.38–1.29)	0.251	0.87 (0.48–1.56)	0.652
	LMICs	0.47 (0.40–0.56)	<0.001	0.70 (0.57–0.87)	0.001
	UMICs	0.57 (0.48–0.68)	<0.001	1.49 (1.22–1.82)	<0.001
	HICs (Ref.)	1		1	
**COVID-19 interrupted substance addiction care**	Do not receive care (Ref.)	1		1	
	Not at all	0.77 (0.61–0.98)	0.031	0.66 (0.51–0.84)	0.001
	Somewhat	0.89 (0.65–1.22)	0.470	0.70 (0.51–0.97)	0.031
	A lot	1.75 (1.05–2.91)	0.032	1.17 (0.68–2.02)	0.568
**Job loss or laid off**	Yes	1.26 (1.02–1.56)	0.030	1.32 (1.05–1.65)	0.018
	No (Ref.)	1		1	
**Lost or reduced wages**	Yes	1.52 (1.29–1.79)	<0.001	1.52 (1.26–1.83)	<0.001
	No (Ref.)	1		1	
**Investment/retirement loss**	Yes	1.01 (0.78–1.30)	0.964	1.45 (1.10–1.92)	0.008
	No (Ref.)	1		1	

AOR: adjusted odds ratio. LICs: low-income countries. LMICs: lower middle-income countries. UMICs: upper middle-income countries. HICs: high-income countries.

Residing in LMICs (AOR=0.70; 95% CI: 0.57–0.87), and having addiction care only interrupted ‘not at all’ (AOR=0.66; 95% CI: 0.51–0.84) or ‘somewhat’ (AOR=0.70; 95% CI: 0.51–0.97), were protective against increased tobacco use. In contrast, residing in UMICs (AOR=1.49; 95% CI: 1.22–1.82), reporting job loss (AOR=1.32; 95% CI: 1.05–1.65), experiencing loss or reduced wages (AOR=1.52; 95% CI: 1.26– 1.83) and loss of investment and retirement benefits (AOR=1.45; 95% CI: 1.10–1.92) were associated with greater odds of tobacco use during the current COVID-19 pandemic.

## DISCUSSION

The current cross-sectional study examined participants across 105 countries that experienced changes in alcohol drinking and tobacco use during the COVID-19 pandemic. It determined the degree to which various factors were associated with self-reported increase in use-levels. This study underscores several important findings. First, an increase in alcohol drinking and tobacco use was reported by a considerable number of users during the first wave of COVID-19 pandemic. Second, increases in consumption varied by country income-level. Residents of LMICs and UMICs had significantly lower odds of reporting an increase in alcohol drinking than residents of HIC. But only residents of LMICs had significantly lower odds of reporting an increase in tobacco use when compared with residents in HICs. Third, treatment-seeking tobacco and alcohol consumers reported interruptions to the care they received for substance dependence during the COVID-19 pandemic, and attending addiction treatment services without interruption during the pandemic acted as a protective factor. Fourth, respondents who lost their jobs and experienced lost/reduced wages had greater likelihood of increased alcohol drinking and tobacco use. Fifth, men and those who experienced loss of investment or retirement benefits reported an increase in tobacco use only.

Similar to the findings of this study, earlier reports from high-income countries also indicated a substantial rise in alcohol drinking and tobacco use since the COVID-19 crisis^6,10,17,24^. This suggests that meeting the goal of a 10% decrease in the consumption of harmful substances by 2025, as suggested by the Global Monitoring Framework for Non-Communicable Diseases, could be extremely challenging and may require public health practitioners and policymakers to implement additional strategies^25,26^. The current study indicates that living in MICs (LMICs and UMICs) in comparison to HICs is protective of increased alcohol drinking and tobacco use during the current pandemic. This could be attributed to decrease purchasing power of people residing in LMICs and UMICs in comparison to those living in HICs due to greater financial stress experienced throughout the pandemic^27-29^. Also, limited access to per capita funds may have reduced the in-country trading of alcohol and tobacco in MICs^30^. Further, it was indicated in a report that the economic toll of COVID-19 pandemic was worse for MICs than HICs^31^. Therefore, this change in trading along with greater financial stress at the household level during the current pandemic may have acted as a barrier for purchasing alcohol and tobacco for residents in MICs^32,33^.

A key relationship between job loss and increase alcohol drinking and tobacco use during the COVID-19 pandemic was detected. Findings show that those who experienced job loss had greater likelihood of consuming more alcohol and tobacco. It is possible that increase in alcohol drinking and tobacco use is a way of coping with the stress associated with job and financial loss^34^. Though psychological distress resulting from job loss may lead to an increase in alcohol drinking and tobacco use, less disposable income and being unable to afford alcohol and tobacco may lead to lower consumption levels^35-37^. Thus, this relationship needs to be explored further.

Next, the current study indicated that the disruptions to addiction treatment services during the pandemic resulted in almost twice the likelihood of an increase in alcohol drinking. There is ample evidence suggesting that alcohol dependence is a chronic relapsing disorder that often requires psychological counselling, behavioral treatment, and pharmacotherapy for more successful outcomes^38-40^. Relapse can occur if treatment services are interrupted^41^. Therefore, disruption of addiction treatment services during the COVID-19 pandemic may have contributed to the increase in alcohol drinking seen in this study. Earlier research also found that 14% of young adults and 17% of mature adults, who were substance-dependent, had reported that their treatment was affected during the current pandemic^13^. It is also possible that the impact of lockdowns on treatment services may have been more severe in some countries than in others^42^. It is, however, challenging to explain why a major disruption in service access may have led to a significant increase in alcohol drinking and not a significant increase in tobacco use, as shown in this study.

Although sex was associated with increase in alcohol drinking and tobacco use, the findings of this study indicate that the sex differences for alcohol drinking and tobacco use were not similar. More males than females had reported increased tobacco use during the current pandemic, but there was no significant relationship between sex and consumption of alcohol. Earlier studies indicate that men drink and smoke more than women in different societies and cultures, irrespective of the situation^43-46^. The reason for this shift and how this shift in male and female alcohol drinking and tobacco use during the pandemic happens to be different could be investigated using qualitative studies.

### Strengths and limitations

While the findings of the current study are unique, limitations should be considered. The cross-sectional nature of the study limits the capacity to confirm any temporal sequence of events and thereby fails to infer causality. Longitudinal studies with probability sampling design may generate more generalizable findings in comparison to the current study. The sampling technique in this study is subject to response bias as only those with access to the internet were able to participate in the survey. Further, the data on alcohol drinking and tobacco use were self-reported and future work should consider using more objective measures of substance use and dependence. Despite the shortcomings, these findings are important considering the challenges in designing and implementing such a large-scale study amidst a pandemic-related global lockdown. This is one of few studies to provide extensive, global-level data on factors linked to measured increases in alcohol drinking and tobacco use during the first wave of the COVID-19 pandemic. The large, representative sample, used in this study included participants from 105 nations with diverse cultures, policies, and social norms. This contributes to the power, generalizability, and significance of these findings. Additionally, this study considered country income levels and other sociodemographic characteristics as covariates in the analyses.

## CONCLUSIONS

This global survey alludes to the unintended consequences of the current COVID-19 pandemic on alcohol drinking and tobacco use. Some proportion of people reported an increase in alcohol drinking and tobacco use during the first wave of the pandemic. Country income-level, financial stress, job loss, and interruption of addiction treatment services were important risk indicators associated with increase in alcohol drinking and tobacco use among users. Therefore, it is critical that the strategies for emergency responses should include support to ameliorate the impact of financial distress and disruption in substance dependence treatment services.

## Data Availability

The data supporting this research are available from the authors on reasonable request.
