# Supplementary material for: Factors associated with an increase in tobacco use and alcohol drinking during the COVID-19 pandemic: A cross-sectional study of data from 105 countries
Source: Tob Induc Dis. 2023 Jan 27;21:14. doi: 10.18332/tid/157205 (PMC9881585; doi:10.18332/tid/157205)
Supplement: Supplementary file 1 [file TID-21-14-s1.pdf]

Appendix-1: Number of participants by country name and income-level.

Country	Income level	N	Country	Income level	N
Burkina Faso	LIC	3	Lebanon	UMIC	8
Congo, Democratic Republic	LIC	5	Libya	UMIC	12
Ethiopia	LIC	7	Malaysia	UMIC	15
Gambia	LIC	9	Mexico	UMIC	550
Liberia	LIC	17	Namibia	UMIC	18
Malawi	LIC	19	Paraguay	UMIC	6
Mali	LIC	26	Peru	UMIC	105
Rwanda	LIC	11	Russia	UMIC	9
Sierra Leone	LIC	8	Serbia	UMIC	37
Sudan	LIC	185	South Africa	UMIC	510
Uganda	LIC	64	Thailand	UMIC	30
Algeria	LMIC	13	Turkey	UMIC	69
Bangladesh	LMIC	17	Australia	HIC	48
Benin	LMIC	10	Austria	HIC	7
Bolivia	LMIC	6	Bahrain	HIC	14
Cambodia	LMIC	5	Belgium	HIC	11
Cameroon	LMIC	11	Canada	HIC	150
Congo, Republic of the	LMIC	13	Chile	HIC	124
Cote d'Ivoire	LMIC	24	Cyprus	HIC	6
Egypt	LMIC	654	Czechia	HIC	3
Eswatini (formerly Swaziland)	LMIC	4	Denmark	HIC	4
Ghana	LMIC	307	Finland	HIC	391
India	LMIC	648	France	HIC	27
Kenya	LMIC	35	Germany	HIC	48
Mongolia	LMIC	7	Greece	HIC	13

Morocco	LMIC	8	Hungary	HIC	194
Myanmar (formerly Burma)	LMIC	9	Iceland	HIC	3
Nepal	LMIC	4	Ireland	HIC	22
Nigeria	LMIC	3964	Israel	HIC	10
Pakistan	LMIC	1547	Italy	HIC	17
Philippines	LMIC	514	Japan	HIC	6
Senegal	LMIC	8	Lithuania	HIC	28
Tanzania	LMIC	20	Mauritius	HIC	7
Tunisia	LMIC	3	Netherlands	HIC	43
Ukraine	LMIC	14	New Zealand	HIC	19
Vietnam	LMIC	5	Norway	HIC	3
Zambia	LMIC	4	Oman	HIC	3
Zimbabwe	LMIC	41	Poland	HIC	6
Argentina	UMIC	500	Portugal	HIC	17
Armenia	UMIC	3	Qatar	HIC	22
Belarus	UMIC	14	Romania	HIC	7
Bosnia and Herzegovina	UMIC	236	Saudi Arabia	HIC	803
Botswana	UMIC	27	Singapore	HIC	3
Brazil	UMIC	100	Slovenia	HIC	3
Bulgaria	UMIC	3	South Korea	HIC	6
China	UMIC	30	Spain	HIC	37
Colombia	UMIC	56	Sweden	HIC	12
Ecuador	UMIC	12	Switzerland	HIC	9
Indonesia	UMIC	55	United Arab Emirates	HIC	132
Iraq	UMIC	9	United Kingdom (UK)	HIC	685
Jordan	UMIC	572	United States of America	HIC	698
Kazakhstan	UMIC	7	Uruguay	HIC	5
Kosovo	UMIC	11			

© 2023 Quadri M.F.A. et al.
